# The causal association between iron status and the risk of autism: A Mendelian randomization study

**DOI:** 10.3389/fnut.2022.957600

**Published:** 2022-11-03

**Authors:** Li Chen, Xingzhi Guo, Chen Hou, Peng Tang, Xin Zhang, Li Chong, Rui Li

**Affiliations:** ^1^Department of Geriatric Neurology, Shaanxi Provincial People’s Hospital, Xi’an, Shaanxi, China; ^2^Shaanxi Provincial Clinical Research Center for Geriatric Medicine, Xi’an, Shaanxi, China; ^3^Institute of Medical Research, Northwestern Polytechnical University, Xi’an, Shaanxi, China

**Keywords:** iron, transferrin, ferritin, autism, Mendelian randomization

## Abstract

Emerging evidence indicates a connection between serum iron levels and autism, but the underlying causal association is yet unclear. Thus, we performed two-sample Mendelian randomization (MR) analysis to evaluate the causal link between iron status on autism, using genetic instruments (*p* < 5E–08) strongly associated with iron status (*N* = 48,972), including serum iron, ferritin, transferrin levels, and transferrin saturation. Summary statistics of autism was obtained from two independent studies conducted by Psychiatric Genomics Consortium (PGC, *N*cases = 5,305, *N*controls = 5,305) and FinnGen Consortium (FC, Round six, *N*cases = 344, *N*controls = 258,095), respectively. Using the inverse-variance weighted (IVW) method, the combined results of PGC and FC demonstrated that genetically determined serum transferrin level was significantly associated with an increased risk of autism [odds ratio (OR) = 1.16, 95% CI: 1.03–1.30, *p* = 0.013]. There was no significant causal effect of serum iron (OR = 0.99, 95% CI: 0.72–1.37, *p* = 0.951), ferritin (OR = 0.88, 95% CI: 0.47–1.64, *p* = 0.676), and transferrin saturation (OR = 0.89, 95% CI: 0.72–1.09, *p* = 0.252) on autism. No obvious pleiotropy was found in this MR study. Taken together, our findings highlight that elevation of serum transferrin level might be associated with a high risk of autism, suggesting a potential role of iron deficiency in autism development. Future studies are warranted to clarify the underlying mechanism, which will pave a new path for the prevention and treatment of autism.

## Introduction

Autism, also called autism spectrum disorder (ASD), is a mental disorder characterized by challenges with communication, social skills, speech, and repetitive behaviors. Epidemic data from the Center for Disease Control and Prevention (CDC) show that autism affects about one in 44 children in the United States (1), and the signs of autism often appear as early as 24–36 months. Although currently, the etiology of autism remains largely unknown, it is suggested that genetic background, environmental factors, and their interaction play a vital role in the development of autism (2). The imbalance of trace elements, such as iron, copper, zinc, selenium, nickel, manganese, aluminum, etc., has been reported to be tightly associated with autism risk (3).

Iron is the most important trace element in maintaining the body’s metabolic process and intracellular oxygen transport. There are about 1.2 billion people who suffer from iron deficiency, especially in children and women (4). In the central nervous system, iron exerts a crucial role in regulating neurotransmitter synthesis, myelination, and neuro-inflammatory response (5). Previous studies indicated that, in autistic children, serum iron and ferritin levels were significantly decreased and increased, respectively. However, a recent meta-analysis with a large sample size demonstrated that there was no significant association between serum ferritin as well as hair iron and autism. Although iron supplementation is a common intervention for the prevention and treatment of various diseases caused by iron deficiency (6), it is also worthy to note that excess iron could increase the levels of reactive oxygen species, contributing to the oxidative damage of DNA, proteins, and lipids, which eventually lead to cell death, also known as ferroptosis (7, 8). Iron supplementation for healthy individuals may pose unexpected health risks owing to the toxicity of iron deficiency and iron overload. Thus, it invokes the need to confirm the causal effect of iron deficiency on autism, which is crucial for the prevention of autism *via* iron supplementation.

The Mendelian randomization (MR) analysis, treating germline genetic variants as instrumental variables (IVs), is now widely used in determining the potential causal link between exposures and corresponding outcomes. MR can effectively minimize the impact of confounding factors that might influence both exposure and outcome phenotype. Therefore, we here performed a two-sample MR analysis to examine whether genetically determined iron status was causally associated with autism development.

## Materials and methods

### Study design and instrumental variants selection

Single nucleotide polymorphisms (SNPs) strongly associated with iron status (*p* < 5E–08) were selected as IVs, which were obtained from genome-wide association studies (GWAS) summary statistics of European ancestry performed by Benyamin et al. (9). The iron status (*N* = 48,927) was indicated in four stages, including serum iron, ferritin, transferrin levels, and transferrin saturation (Supplementary Table 1). Instrument variables were clumped based on the 1,000 Genomes Project linkage disequilibrium (LD) structure (r^2^ < 0.001 within 10,000 kb, European). The detailed information on study design has been well-described in the original publication (9).

### Autism genome-wide association studies datasets

Two independent GWAS datasets on autism were obtained from studies performed by the Psychiatric Genomics Consortium (PGC) and the FinnGen Consortium (Round 6),^1^ respectively. For summary statistics from PGC, a total of 5,305 autism cases and 5,305 controls of European descent were included (OpenGWAS: ieu-a-1184) (10). For summarized association data from FinnGen Consortium (Round 6), there were 344 cases and 258,095 normal controls of European descent. To further validate the results, we also adopted the latest autism GWAS dataset of Integrative Psychiatric Research and PGC (iPSYCH-PGC) as a replication (*N*cases = 18,382, *N*controls = 27,969, OpenGWAS: ieu-a-1185) (11), which contained GWAS summary statistics from population-based samples of iPSYCH and family-based samples of PGC (10). Autism was diagnosed according to Autism Diagnostic Interview-Revised (ADI-R) and/or Autism Diagnostic Observation Schedule (ADOS) (12).

### Mendelian randomization analysis

**FIGURE 1:**
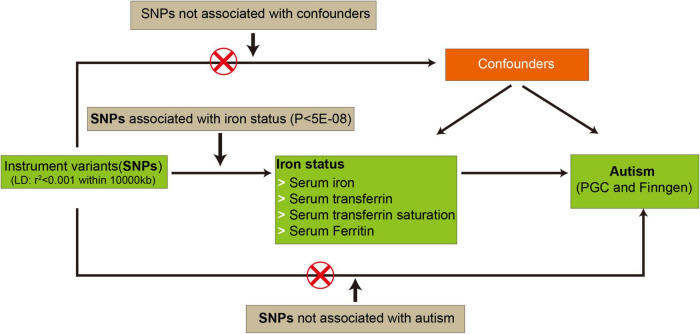
The flowchart in this two-sample Mendelian randomization study. The red cross means that genetic variants are not associated with confounding factors and outcomes. PGC, psychiatric genomics consortium; SNPs, single nucleotide polymorphisms.

Mendelian randomization analysis was conducted using the TwoSampleMR package (v0.5.6) (13). Moreover, if SNPs selected as IVs were absent in the summary statistics of autism, overlapping proxy SNPs in LD (r^2^ = 0.8) were used instead. To strengthen the reliability of MR analysis, SNPs with minor allele frequency (MAF) less than 0.3 were also removed. The inverse-variance weighted (IVW), providing a robust causal evaluation under a lack of directional pleiotropy, was employed as the primary method to calculate causal estimates between iron status and autism. Meanwhile, both weighted median and MR–Egger regression methods were also applied to assess the causal estimates. After MR analysis, a meta-analysis was performed to combine the overall causal estimates from PGC or iPSYCH-PGC and FinnGen Consortium, respectively. A *p*-value less than 0.05 was considered statistically significant. Three corresponding principal assumptions and the flowchart in this study was described in Figure 1.

### Pleiotropy, heterogeneity, and power analysis

Mendelian randomization-Pleiotropy RESidual Sum and Outlier (MR-PRESSO) test with default parameters was performed to identify horizontal pleiotropic outliers (14), and the MR-Egger intercept test was conducted to assess whether there was potential horizontal pleiotropy driving the MR results. Meanwhile, the IVW method and MR-Egger regression were also used to estimate heterogeneity, which was quantified by Cochran Q statistics. To validate the reliability of the causal association between iron status and autism, we performed a power analysis using the online tool mRnd^2^ (15). F values of more than 10 indicated good stability.

## Results

### Summary of the Mendelian randomization analysis

A detailed list of all harmonized instrumental variables, namely SNPs, for each exposure-outcome group was archived in the Supplementary Data Sheet 1. The causal estimates (OR values and 95% CI) of the IVW with *p*-values, number of SNPs, and combined estimates of the meta-analysis were presented in forest plots. The results of MR-PRESSO global with *p*-values, MR-Egger intercept with *p*-values, heterogeneity in IVW, and MR Egger test for each exposure-outcome pair were shown in Table 1 and Supplementary Table 2. Causal estimates using weighted median and MR–Egger method were also calculated.

**TABLE 1:** Heterogeneity and power analysis of iron status on autism.

Exposure\Outcome	Method	PGC (autism)				FinnGen consortium (autism)			
			
		MR-egger intercept (*P*)	Cochran-Q (*P*)	F-statistic	MR_PRESSO (*P*)	MR-egger intercept (*P*)	Cochran-Q (*P*)	F-statistic	MR_PRESSO (*P*)
Iron (μmol/l)	MR-Egger	0.044 (0.495)	6.64 (0.035)	368.72	11.96 (0.189)	−0.007 (0.939)	1.84 (0.397)	8957.97	3.06 (0.713)
	IVW		8.91 (0.030)				1.85 (0.603)		
Transferrin (g/l)	MR-Egger	−0.028 (0.109)	0.72 (0.993)	1191.06	7.94 (0.659)	0.012 (0.780)	1.67 (0.946)	28988.48	3.12 (0.952)
	IVW		4.24 (0.751)				1.76 (0.971)		
Transferrin saturatio*n* (%)	MR-Egger	−0.033 (0.446)	4.38 (0.111)	741.80	8.41 (0.240)	−0.091 (0.325)	0.08 (0.956)	18045.50	3.72 (0.602)
	IVW		6.31 (0.097)				1.75 (0.625)		
Ferritin (log10, μg/l)	MR-Egger	0.001 (0.978)	14.39 (0.002)	112.84	18.87 (0.053)	−0.052 (0.600)	0.44 (0.930)	2725.23	1.57 (0.915)
	IVW		14.40 (0.006)				0.78 (0.940)		

MR, Mendelian randomization; PGC, psychiatric genomics consortium; IVW, inverse-variance weighted; MR_PRESSO, Mendelian randomization pleiotropy RESidual sum and outlier; *p*, *p*-value.

### Causal effects of iron status on autism

Using the IVW method, genetically predicted serum transferrin was significantly associated with an increased risk of autism in PGC (OR = 1.16, 95% CI: 1.03–1.31, *p* = 0.018), but not FinnGen Consortium (OR = 1.15, 95% CI: 0.80–1.65, *p* = 0.451). A further meta-analysis based on the above two independent data showed that serum transferrin remained associated with an increased risk of autism (OR = 1.16, 95% CI: 1.03–1.30, *p* = 0.013). Similar results were found using both weighted median (OR = 1.179, 95% CI: 1.043–1.333) and MR Egger (OR = 1.207, 95% CI: 0.939–1.553). No obviously directional horizontal pleiotropy and heterogeneities were found in MR-PRESSO global (RSSobs = 7.94, *p* = 0.659) and MR-Egger Intercept test (*p* = 0.109). Additionally, there was no evidence showing significant heterogeneity in MR Egger (Cochran Q = 0.72, *p* = 0.993) and IVW (Cochran Q = 4.24, *p* = 0.751) regression test.

**FIGURE 2:**
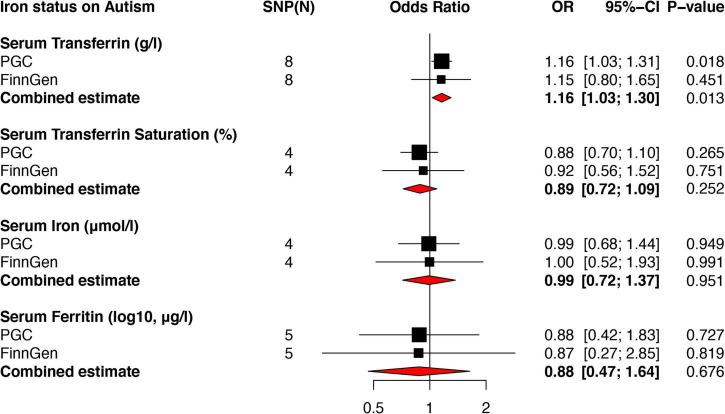
Forest plots of MR analyses showing the causal effects of iron status on autism using the IVW method. Two independent autism genome-wide association studies (GWAS) datasets from PGC and FinnGen Consortium were used to evaluate the causal effect. The combined estimates were presented as red diamonds. PGC, psychiatric genomics consortium; IVW, inverse-variance weighted; MR, Mendelian randomization.

**FIGURE 3:**
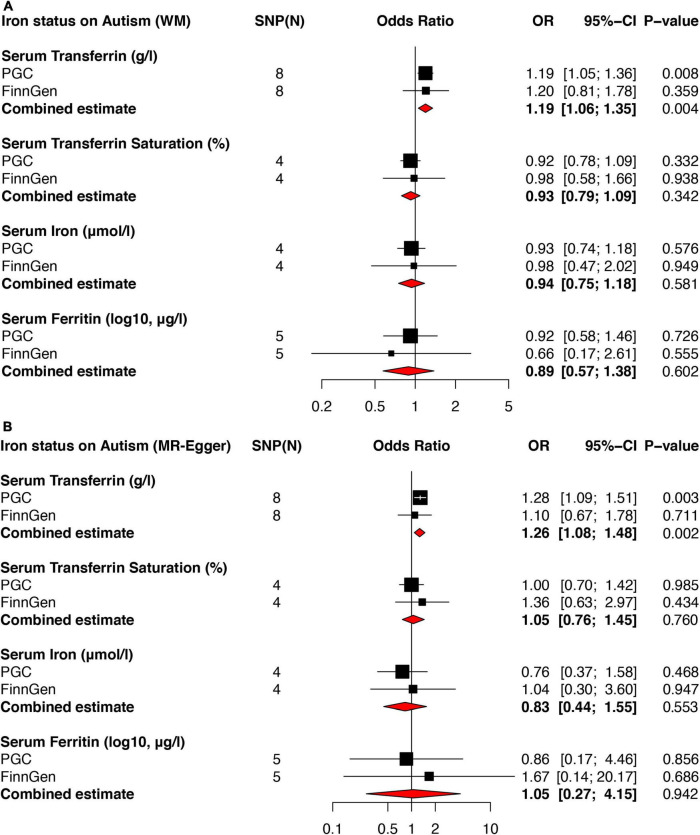
Forest plots of MR analyses showing the causal effects of iron status on autism using weighted median and MR Egger method. Two independent autism GWAS datasets from PGC and FinnGen Consortium were used to evaluate the causal effect. The combined estimates were presented as red diamonds. **(A)** Showed the causal effect of iron status on autism using the weighted median method. **(B)** Showed the causal effect of iron status on autism using the MR Egger method. PGC, psychiatric genomics consortium; WM, weighted median; MR, Mendelian randomization.

Using the IVW method, no significantly causal link between serum iron and ferritin levels and transferrin saturation on autism was observed in both PGC and FinnGen Consortium datasets (Figure 2). Further meta-analysis combining the causal estimates of PGC and FinnGen Consortium also showed that there was no association between serum iron (OR = 0.99, 95% CI: 0.72–1.37, *p* = 0.951), ferritin (OR = 0.88, 95% CI: 0.47–1.64, *p* = 0.676) as well as transferrin saturation (OR = 0.89, 95% CI: 0.72–1.09, *p* = 0.252) and autism. Similar results were found using weighted median (Figure 3A) and MR Egger methods (Figure 3B). No obvious horizontal pleiotropy and heterogeneity were found in the MR analysis between serum iron, transferrin, and transferrin saturation and autism. To further validate the MR results above, we replicated the MR analysis using the latest GWAS dataset of iPSYCH-PGC, which showed consistent results (Supplementary Figure 1).

### Power analysis

The overall F statistics for genetic variants selected as IVs of serum iron, ferritin, and transferrin levels and transferrin saturation in PGC were 368.72, 112.84, 1191.06, and 741.80 individually. For FinnGen Consortium, the F statistics of serum iron, ferritin, and transferrin levels and transferrin saturation in were 8957.97, 2725.23, 28988.48, and 18045.50. The F value from the iPSYCH-PGC dataset also showed that there was no obvious weak instrumental bias (Supplementary Table 2).

## Discussion

Although various studies have been performed to explore the association between iron status and autism, this study here is the first MR analysis conducted to evaluate the naturally causal link between iron status on autism susceptibility. Using the IVW method, our results demonstrated that genetically predicted serum transferrin was causally associated with an increased risk of autism, indicating that transferrin may be a potential marker for autism development.

Iron deficiency could cause numerous detrimental effects in people, such as anemia, fatigue, atrophic glossitis, hair loss, etc. (16), and is also associated with the risk of many diseases. For example, previous studies showed that iron deficiency was associated with an increased risk of type 2 diabetes, Parkinson’s disease, and cancers (17–20). Maternity with a lower mean daily iron intake led to an increased risk of autism in offspring (21). Numerous studies have suggested that iron deficiency was a common phenomenon in autism (22), and iron supplementary has been widely advised for pregnant women and children with finicky habitation. However, it is also hypothesized that excess iron intake might contribute to a higher susceptibility to autism *via* over-activating the immune system (23). Thus, it is urgent for clinicians to find an optimistic marker to guide iron supplementation, especially for those who do not need iron supplementation. There are four classical iron biological states, including serum iron, ferritin, transferrin levels, and transferrin saturation, commonly used in reflecting iron status in humans. In this study, we found that only serum transferrin was associated with an increased risk of autism, but not serum iron, ferritin, and transferrin saturation. Indeed, a recent meta-analysis also showed that hair iron and serum ferritin were not significantly different between controls and autism patients (24). Meanwhile, serum transferrin levels were significantly decreased in autistic children as compared to their non-autistic siblings (25). These data suggested that transferrin, but not iron levels might be a more sensitive and efficient marker for autism development.

The main function of transferrin, an iron-binding glycoprotein, is to mediate the transport of free iron to suitable cells with corresponding surface receptors around the body (26). Transferrin plays a vital role in transporting iron to erythroid precursors in the bone marrow, maturity dysfunction of which would cause anemia (27). Increased serum transferrin level is often observed in people with iron deficiency anemia, which is tightly associated with autism and other neurological diseases (28). It is suggested that transferrin saturation could also serve as an effective diagnostic criterion for iron deficiency (29). In addition, transferrin also was reported to have an anti-oxidative function, which could further reduce the risk of autism (30). Due to the elevation of transferrin often happening earlier than the reduction of serum iron, it might contribute to a positive association between autism and serum transferrin, but not iron.

Previous studies suggest that immune dysfunction also plays a vital role in the development of autism (31). The over-activated neuro-inflammatory response was found in the brain specimen of both children and adults with autism (32). In addition, the plasma and cerebral-spinal fluid pro-inflammatory factors were also significantly increased in autistic patients (33). Yet interestingly, it is reported that transferrin also exerts an important role in regulating the immune response in the brain. Treatment of transferrin-derived synthetic peptide could induce a pro-inflammatory phenotype of macrophage, which might contribute to an elevated cytokines level (34). A study in fish also suggested that transferrin could activate macrophages in response to pathogens (35). Taken together, these data suggested that iron deficiency might not directly increase the pathological changes in autism, and might contribute to a higher risk of autism indirectly *via* elevating the expression of transferrin. Similarly, our results showed that only transferrin was significantly associated with autism risk, but not serum iron levels. Thus, a current challenge for researchers is to determine the underlying mechanisms contributing to the association between an elevation of transferrin and autism risk, which is important for establishing prevention strategies in patients with autism susceptibility.

There are several obvious strengths in our study. No pleiotropy were found in the MRPRESSO test and MR-Egger Intercept test, suggesting the good reliability of our results. Moreover, the causal estimates obtained from three different methods (IVW, MR Egger, and weighted median) were consistent. Finally, although the MR results of serum transferrin using the latest autism GWAS dataset of iPSYCH-PGC were not statistically significant, the causal estimates showed a similar trend and were significant in the meta-analysis. Meanwhile, the results of the meta-analysis showed that genetically determined serum transferrin was still significantly associated with an increased risk of autism, even if using the negative result from FinnGen Consortium, indicating our results were robust.

Owing to the intrinsic principles and assumptions behind MR, the results of the current study should be interpreted cautiously and some limitations should also be addressed here. First, less than ten SNPs were selected as IVs for all four traits of iron status, which might weaken the association between iron status and autism. Second, although the combined estimates of the meta-analysis showed serum transferrin was associated with an increased risk of autism, the causal effect obtained from FinnGen Consortium was not statistically significant. Third, there were only 344 autism cases from the GWAS of the FinnGen Consortium. Further released data with larger case samples from FinnGen Consortium are required to verify the associations above. Forth, all genetic data used in this MR study are from the European population. Whether the causal link between iron status and autism observed in this study is common in other ethnic groups needs to be further verified.

## Conclusion

In summary, the results of this MR study demonstrate that genetically predicted serum transferrin levels might be causally associated with an increased risk of autism. Our findings reminded clinicians that more attention and concern should be paid to iron status in children with autism family history. To clarify the underlying mechanism of iron deficiency contributing to autism susceptibility is vital for guiding autism prevention and therapy.

## Data availability statement

The original contributions presented in this study are included in the article/Supplementary material, further inquiries can be directed to the corresponding authors. All R scripts applied in MR analysis are available from the authors upon request.

## Author contributions

LChe, XG, and RL conceived and designed the project. LChe, XG, CH, and XZ collected and analyzed the data and drafted the manuscript. PT, LCho, and RL revised the manuscript. All authors have read and approved the final manuscript.

## Footnotes

1
https://finngen.gitbook.io/documentation

2
http://cnsgenomics.com/shiny/mRnd/

## Supplementary material

Supplementary Data Sheet 1 Characteristics of harmonized instrumental variables used in Mendelian randomization (MR) analysis between iron status and autism.
