# Supplementary material for: The causal association between iron status and the risk of autism: A Mendelian randomization study
Source: Front Nutr. 2022 Nov 3;9:957600. doi: 10.3389/fnut.2022.957600 (PMC9669792; doi:10.3389/fnut.2022.957600)
Supplement: Supplementary file 1 [file Table_1.DOCX]

Supplementary Table S1. Summary of the SNPs selected as instrumental variants for iron status*.

| **SNP** | **Chr** | **Pos** | **Alt** | **Ref** | **EAF** | **Beta** | **SE** | **Pval** | **N** | **Phenotype** |
| --- | --- | --- | --- | --- | --- | --- | --- | --- | --- | --- |
| rs8177240 | 3 | 133477701 | T | G | 0.669 | -0.066 | 0.007 | 6.65E-20 | 48972 | Serum iron (μmol/l) |
| rs1800562 | 6 | 26093141 | A | G | 0.067 | 0.328 | 0.016 | 2.72E-97 | 48972 | Serum iron (μmol/l) |
| rs7385804 | 7 | 100235970 | A | C | 0.621 | 0.064 | 0.007 | 1.36E-18 | 48972 | Serum iron (μmol/l) |
| rs855791 | 22 | 37462936 | A | G | 0.446 | -0.181 | 0.007 | 1.32E-139 | 48972 | Serum iron (μmol/l) |
| rs744653 | 2 | 190378750 | T | C | 0.854 | 0.068 | 0.01 | 1.35E-11 | 48972 | Serum transferrin (g/l) |
| rs8177240 | 3 | 133477701 | T | G | 0.669 | -0.38 | 0.007 | 0 | 48972 | Serum transferrin (g/l) |
| rs9990333 | 3 | 195827205 | T | C | 0.46 | -0.051 | 0.007 | 1.95E-13 | 48972 | Serum transferrin (g/l) |
| rs1800562 | 6 | 26093141 | A | G | 0.067 | -0.479 | 0.016 | 8.90E-196 | 48972 | Serum transferrin (g/l) |
| rs4921915 | 8 | 18272466 | A | G | 0.782 | 0.079 | 0.009 | 7.05E-19 | 48972 | Serum transferrin (g/l) |
| rs6486121 | 11 | 13355770 | T | C | 0.631 | -0.046 | 0.007 | 3.89E-10 | 48972 | Serum transferrin (g/l) |
| rs174577 | 11 | 61604814 | A | C | 0.33 | 0.062 | 0.007 | 2.28E-17 | 48972 | Serum transferrin (g/l) |
| rs855791 | 22 | 37462936 | A | G | 0.446 | 0.044 | 0.007 | 1.98E-09 | 48972 | Serum transferrin (g/l) |
| rs8177240 | 3 | 133477701 | T | G | 0.669 | 0.1 | 0.008 | 7.24E-38 | 48972 | Serum transferrin saturation (%) |
| rs1800562 | 6 | 26093141 | A | G | 0.067 | 0.577 | 0.016 | 2.19E-270 | 48972 | Serum transferrin saturation (%) |
| rs7385804 | 7 | 100235970 | A | C | 0.621 | 0.054 | 0.008 | 6.07E-12 | 48972 | Serum transferrin saturation (%) |
| rs855791 | 22 | 37462936 | A | G | 0.446 | -0.19 | 0.008 | 6.41E-137 | 48972 | Serum transferrin saturation (%) |
| rs744653 | 2 | 190378750 | T | C | 0.854 | -0.089 | 0.01 | 8.37E-19 | 48972 | Serum log10 ferritin (μg/l) |
| rs1800562 | 6 | 26093141 | A | G | 0.067 | 0.204 | 0.016 | 1.54E-38 | 48972 | Serum log10 ferritin (μg/l) |
| rs651007 | 9 | 136153875 | T | C | 0.202 | -0.05 | 0.009 | 1.31E-08 | 48972 | Serum log10 ferritin (μg/l) |
| rs411988 | 17 | 56709034 | A | G | 0.564 | -0.044 | 0.007 | 1.59E-10 | 48972 | Serum log10 ferritin (μg/l) |
| rs855791 | 22 | 37462936 | A | G | 0.446 | -0.055 | 0.007 | 1.38E-14 | 48972 | Serum log10 ferritin (μg/l) |

Note: SNP, Single nucleotide polymorphisms; Chr, Chromosome; Pos, Position; Alt, Alternative allele; Ref, Reference allele; EAF, Effect allele frequency; Beta, Effect of Alt allele; SE, Standard error; Pval, P-value; N, Sample size.

*: Summarized by Benyamin et al. (PMID: 25352340).
