# Supplementary material for: The causal association between iron status and the risk of autism: A Mendelian randomization study
Source: Front Nutr. 2022 Nov 3;9:957600. doi: 10.3389/fnut.2022.957600 (PMC9669792; doi:10.3389/fnut.2022.957600)
Supplement: Supplementary file 2 [file Table_2.DOCX]

Supplementary Table S2. Heterogeneity and power analysis of iron status on autism (iPSYCH-PGC).

| **Exposure\Outcome** | **Method** |  | **iPSYCH-PGC (autism)** | | | |  |
| --- | --- | --- | --- | --- | --- | --- | --- |
|  |  |  | MR-Egger intercept (P) | Cochran-Q (P) | F-statistic | MR_PRESSO (P) |  |
| **Iron (μmol/l)** | MR-Egger |  | 0.013(0.550) | 3.62(0.162) | 1607.43 | 8.93(0.393) |  |
|  | IVW |  |  | 4.54(0.208) |  |  |  |
| **Transferrin (g/l)** | MR-Egger |  | -0.011(0.188) | 3.66(0.722) | 5199.90 | 6.62(0.666) |  |
|  | IVW |  |  | 5.85(0.556) |  |  |  |
| **Transferrin Saturation (%)** | MR-Egger |  | -0.004(0.773) | 2.67(0.262) | 3237.28 | 5.32(0.529) |  |
|  | IVW |  |  | 2.82(0.419) |  |  |  |
| **Ferritin (log10, μg/l)** | MR-Egger |  | 0.018(0.409) | 5.30(0.150) | 489.59 | 14.14(0.225) |  |
|  | IVW |  |  | 6.92(0.140) |  |  |  |

Note: MR, Mendelian randomization; iPSYCH-PGC, Integrative Psychiatric Research and Psychiatric Genomics Consortium; IVW, inverse-variance weighted; MR_PRESSO, Mendelian Randomization Pleiotropy RESidual Sum and Outlier; P, P value.
