# Supplementary material for: The causal association between iron status and the risk of autism: A Mendelian randomization study
Source: Front Nutr. 2022 Nov 3;9:957600. doi: 10.3389/fnut.2022.957600 (PMC9669792; doi:10.3389/fnut.2022.957600)
Supplement: Supplementary file 3 [file Image_1.PDF]

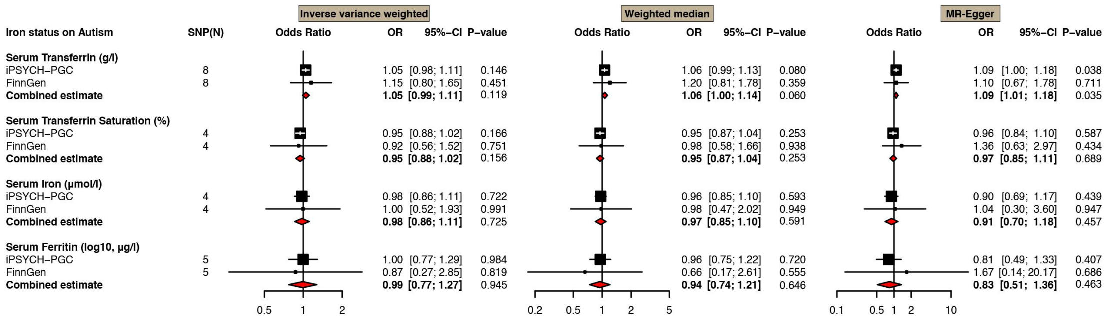

Supplementary Figure S1. Forest plots of MR analyses showing the causal effects of iron status on autism (iPSYCH-PGC) using the IVW, weighted median, and MR-Egger methods. Two independent autism GWAS datasets from iPSYCH-PGC and FinnGen Consortium were used to evaluate the causal effects. The combined estimates were presented as red diamonds. iPSYCH-PGC, Integrative Psychiatric Research and Psychiatric Genomics Consortium; IVW, inverse-variance weighted; OR, odds ratio; MR, Mendelian randomization.
